# Estimation of Soil Organic Matter in Moso Bamboo (*Phyllostachys edulis*) Forests Based on a Synergistic Matching Mechanism Between Feature Selection and Models

**DOI:** 10.3390/s26113515

**Published:** 2026-06-02

**Authors:** Mingxin Li, Zhongyuan Li, Yuzhen Wu, Hanyue Song, Siwen Lin, Yangyang Zhang, Zhihui Yu, Jian Liu, Kunyong Yu

**Affiliations:** 1 College of Forestry, Fujian Agriculture and Forestry University, Fuzhou 350002, China; 52304022112@fafu.edu.cn (M.L.); 12404028004@fafu.edu.cn (Y.W.); 22304030004@fafu.edu.cn (H.S.); 12427096007@fafu.edu.cn (S.L.); 12304030002@fafu.edu.cn (Y.Z.); 52304022035@fafu.edu.cn (Z.Y.); 2 University Key Lab for Geomatics Technology and Optimize Resources Utilization in Fujian Province, Fujian Agriculture and Forestry University, Fuzhou 350002, China; xmlhyuan@163.com; 3 Xiamen Luhengyuan Environmental Planning and Construction Co., Ltd., Xiamen 361000, China

**Keywords:** *Phyllostachys edulis*, soil organic matter, vis-NIR spectroscopy, synergistic adaptation strategy, data imbalance effect

## Abstract

**Highlights:**

**What are the main findings?**

- Preprocessing attenuated color features in limited-band field in situ spectra.
- CARS-SVR effectively avoids high-value underestimation from data imbalance.

**What are the implications of the main findings?**

- RF with physical indices enables low-cost, effective low-content SOM estimation.
- A synergistic approach serves as a reference for forest soil SOM estimation.

**Abstract:**

Rapid and effective estimation of soil organic matter (SOM) is crucial for the scientific management of Moso bamboo forests. This study investigated Moso bamboo forest soils in Yongan City, Fujian Province, and systematically evaluated the synergistic adaptation strategies coupling spectral preprocessing methods, feature extraction strategies, and machine learning models based on visible and shortwave near-infrared (Vis-NIR) spectroscopy. The results indicated that: (1) Conventional preprocessing algorithms attenuated the SOM spectral feature signals dominated by soil color within the limited wavelength range of field in situ spectral data, resulting in a general decline in the accuracy of the estimation models. (2) Feature extraction and modeling algorithms exhibited distinct adaptability across different content intervals. Within the low-content interval (<15 g/kg), simple physical indices combined with random forest (RF) achieved effective estimation at a lower computational cost (RPD = 2.18). Within the high-content interval (>25 g/kg), the synergistic strategy of the CARS algorithm combined with support vector regression (SVR) yielded the optimal estimation performance (R^2^ = 0.83, RPD = 2.45) and effectively mitigated the underestimation of high values caused by data imbalance. In conclusion, this study proposed a feature–model synergistic estimation approach, validating its feasibility for estimating SOM in Moso bamboo forests under the specific constraints of the current study area, thereby serving as a valuable reference for forest soil SOM monitoring in specific regions.

## 1. Introduction

Soil organic matter (SOM) is a critical indicator of soil fertility [1,2], and an essential component of the carbon cycle in terrestrial ecosystems. The effective and accurate estimation of SOM is of global significance for maintaining carbon cycle processes and mitigating climate change [3]. Moso bamboo (*Phyllostachys edulis*), as an integral part of forest ecosystems, is characterized by its wide distribution and rapid growth, sequestering a substantial amount of carbon [4]. In the collective forest regions of central and southern China, Moso bamboo plantations serve not only as a primary economic source for local farmers but also play a pivotal role in sustaining regional carbon sink functions [5]. Therefore, the rapid and precise estimation of SOM in Moso bamboo (*Phyllostachys edulis*) plantations plays a pivotal role in facilitating scientific management, promoting bamboo growth, and sustaining carbon sinks.

Traditional chemical analysis methods for determining SOM content are time-consuming, labor-intensive, costly, and highly destructive, failing to meet the requirements for the rapid monitoring of SOM in Moso bamboo plantations [6,7,8]. Recently, driven by advancements in hyperspectral technology, visible and near-infrared (Vis-NIR) spectroscopy has been widely applied in soil property estimation. This technology has achieved excellent estimation performance for SOM; it not only circumvents the limitations inherent in traditional chemical analyses [9], but also satisfies the current demands for SOM monitoring [10]. However, Moso bamboo (*Phyllostachys edulis*) stands undergo continuous artificial management practices, such as deep plowing, fertilization, and harvesting [11]. Additionally, the unique underground rhizome network of Moso bamboo remains interconnected, facilitating the redistribution of soil organic matter from fertile to barren areas [12]. This regulatory mechanism further increases the complexity of the spatial distribution of SOM in these plantations. The combined effects of long-term intensive management, complex topographic variations, and the nutrient-regulating function of the rhizome network [11,13,14] result in a high degree of spatial heterogeneity of SOM in Moso bamboo stands. This characteristic not only creates complex nonlinear relationships between SOM content and spectral features but also limits the applicability of near-infrared spectroscopy for estimating SOM in these environments [15].

The spectral features of SOM in the visible and near-infrared (Vis-NIR) range are primarily derived from the vibrational responses of hydrogen-containing groups and electron transition effects within organic components, macroscopically manifesting as changes in soil color [16,17,18]. Moso bamboo (*Phyllostachys edulis*) typically grows in the typical red soil regions of southern China. The soils in these regions generally contain high levels of iron oxides [19], such as hematite, and are affected by certain levels of soil moisture. These factors exhibit pronounced absorption features in the near-infrared band, which easily overlap with the inherently weak absorption signals of SOM, thereby affecting SOM estimation [20]. Numerous studies have utilized preprocessing methods to eliminate spectral baseline drift and scattering effects, mitigating the interference from such background and environmental factors [21]. Their results indicate that preprocessing algorithms, such as Standard Normal Variate (SNV) or Multiplicative Scatter Correction (MSC), can effectively enhance the estimation performance and reliability of the models. It should be noted, however, that the effect of spectral preprocessing on SOM estimation depends on multiple factors, such as the spectral range, signal-to-noise ratio (SNR), and soil type; it can either help or hamper the estimation. Studies have demonstrated that improper or excessive application of preprocessing algorithms can lead to the loss of sensitive feature information related to SOM and may even amplify spectral noise in low-reflectance regions [22,23]. For instance, Eslamifar et al. reported that the SNV algorithm negatively affected model stability in soil P_2_O_5_ estimation [24]. Moreover, the impact of various preprocessing algorithms on estimation performance is inconsistent, as not all methods effectively mitigate interference [25]; certain algorithms have even been found to decrease the estimation performance of SVR models in predicting SOM [26]. In scenarios where preprocessing fails to enhance the correlation between soil spectra and SOC, models utilizing preprocessed spectra may yield inferior results compared to those using untreated raw spectra [27].

In addition to the potential negative impacts of preprocessing, collinearity among band variables and data redundancy are also critical factors restricting the estimation performance of soil organic matter [28]. Numerous studies have demonstrated that utilizing algorithms such as CARS, PSO, and ACO to extract feature bands can effectively eliminate the interference of redundant variables and extract feature bands related to SOM. The estimation performance of models constructed using these bands is typically significantly superior to those utilizing full-spectrum data [29,30,31]. To further address the spectral estimation challenges posed by complex, heterogeneous soils, recent studies have begun employing deep learning technologies, such as one-dimensional convolutional neural networks (1D-CNN), to extract deeper spectral features highly correlated with SOC. The estimation performance of these methods often outperforms that of traditional neural networks [32]. Meanwhile, to address the challenges encountered in the cross-regional application of field in situ spectral data in complex environments, recent studies have utilized domain adaptation strategies, such as deep transfer learning (DTL), to predict in situ SOM. In terms of calibration transfer, this method performs significantly better than direct standardization (DS) and its derivative methods (DS-airPLS), substantially enhancing the cross-regional generalization capacity of the models [33]. However, existing methodologies predominantly focus on agricultural soils with relatively uniform textures, lacking research on the highly heterogeneous soils of Moso bamboo (Phyllostachys edulis) plantations. The applicability of existing spectral preprocessing methods, feature band extraction technologies, and various machine learning models to the complex soil characteristics of Moso bamboo (*Phyllostachys edulis*) plantations remains to be systematically investigated. Furthermore, most studies treat feature band extraction and model construction as two independent stages, attributing estimation limitations to spectral data quality or algorithmic selection. This approach overlooks the intrinsic compatibility between underlying spectral features and algorithmic selection. Therefore, to elucidate the synergistic matching mechanism between feature extraction strategies and estimation models, this study focused on the red soil of Moso bamboo (*Phyllostachys edulis*) plantations in Yongan City, Fujian Province. The specific objectives were to: (1) evaluate the impact of preprocessing on the spectral features of SOM in these plantations; (2) construct and compare the estimation performance of models based on simple physical index feature sets, feature sets derived from extraction algorithms such as CARS, and full-band data; and (3) reveal the synergistic matching effects between different feature extraction schemes and model algorithms, clarifying how specific data features adapt to particular algorithmic principles, thereby providing a scientific basis and technical support for the rapid and effective estimation of soil fertility in Moso bamboo plantations.

## 2. Materials and Methods

### 2.1. Study Area

**Figure 1:**
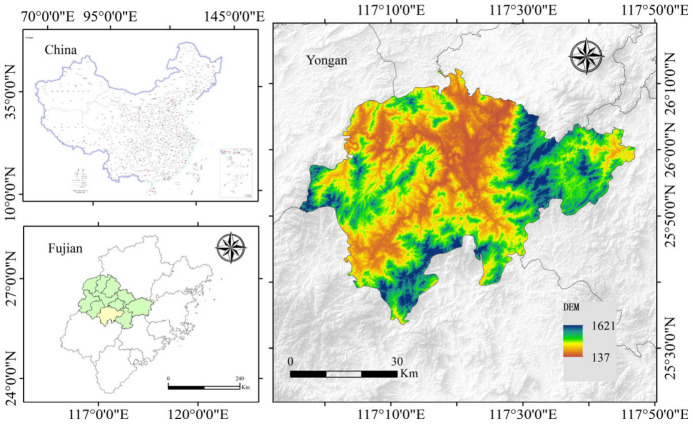
Overview map of the study area.

The study area, Yong’an City, is located in the west-central part of Fujian Province (116°56′–117°47′ E, 25°33′–26°12′ N), measuring 85 km in width from east to west and 72 km in length from north to south, and borders Datian County, Liancheng County, Zhangping City, Mingxi County, and Sanyuan District [34] (Figure 1).The region has an average elevation of 626.45 m and experiences a mid-subtropical maritime monsoon climate, characterised by long summers and short winters, a warm and humid environment with abundant rainfall, a mean annual temperature of 14.3–19.2 °C, and an annual precipitation of 1490–2060 mm [35,36]. The city currently possesses a forest land area of 25.22 × 10^4^ ha with a high forest coverage rate of 82.85%, and a bamboo plantation area of 6.83 × 10^4^ ha, making it an important bamboo resource production region in China [37]. Vegetation types include evergreen broad-leaved forests, temperate coniferous forests, warm coniferous forests, mixed coniferous and broad-leaved forests, bamboo plantations, shrublands, and swamps [36]. The soil types are primarily Red soils and Yellow soils; the Red soils are mainly distributed in suburban areas, while the Yellow soils are predominantly found in the mountainous regions of the east and southwest [35].

### 2.2. Collection of Soil Samples and Determination of Physicochemical Properties

**Figure 2:**
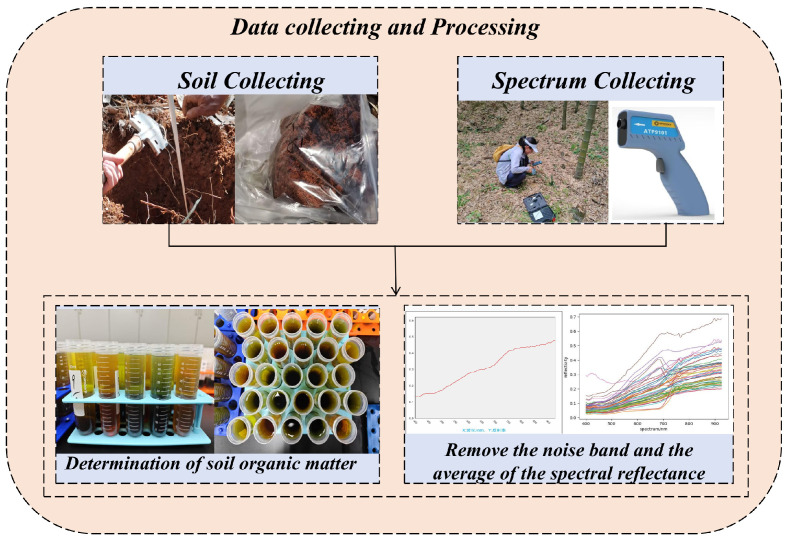
Schematic diagram of data acquisition.

Soil samples were collected in Shangping Township, Yongan City, in July 2024. Under a uniform management regime for the Moso bamboo plantation plots, following a uniform distribution principle that accounted for local topography, land use status, and spatial distribution, plots measuring 30 m × 30 m were established, and three soil sampling points were designated at the upper, middle, and lower slope positions within each plot to represent the sampling units. At each unit, surface soil sub-samples (0–20 cm depth) were collected, weighing approximately 1 kg. These sub-samples were then thoroughly mixed to form a composite soil sample and stored in sealed bags, yielding a total of 144 samples. The soil samples were naturally air-dried indoors; stones, plant roots, and visible humus were removed, after which the samples were ground and passed through a 2 mm sieve. Finally, the SOM content of the samples was determined using the hydration heat-potassium dichromate oxidation-colorimetric method. Based on standard curve calibration and blank controls to eliminate background interference, all samples were measured in triplicate, and the relative standard deviation (RSD) was controlled to within 5%. Following an outlier detection test, 5 anomalous samples were excluded, resulting in 139 valid samples retained for subsequent modeling analysis (Figure 2).

### 2.3. Acquisition of Soil Spectral Data

Spectral data of soil samples were collected using an ATP9101 field spectrometer(Optosky (Xiamen) Photonics Inc., Xiamen, China), which has a wavelength range of 390–926 nm, covering the visible and short-wave near-infrared regions. Spectral collection was conducted between 10:00 and 15:00 on clear, windless days. During measurement, the probe was positioned vertically downward approximately 20 cm from the ground with a 25° field of view, and the resampling interval was 2 nm. A standard white panel calibration was strictly performed prior to measuring each soil sample, and the relative positions of the instrument’s measurement height and the soil sample remained constant before formal measurements began. Spectral curves were collected from four directions for each sampling point by rotating the instrument four times at 90° intervals. After collecting three original spectral reflectance measurements in each direction, the arithmetic mean of all 12 spectra was taken as the full-band original spectral data for that soil sample. In this study, spectral data served as the X variable and SOM content as the Y variable. The sample set partitioning based on joint x-y distance (SPXY) algorithm was used to partition the soil sample set, as this method enhances the variability and representativeness of the samples and improves the stability of the developed models [38]. The basic characteristics and management measures of the corresponding sampling plots are presented in Table 1.

### 2.4. Pre-Processing of Soil Spectral Data and Construction of Feature Sets

#### 2.4.1. Pre-Processing of Soil Spectral Data

To improve the quality of the spectral data, the bands at the edges of the soil sample spectral curves, which are significantly affected by external noise and have a low signal-to-noise ratio, were removed. The spectral data within the 410–915 nm wavelength range were retained for subsequent analysis. The Savitzky–Golay (SG) [39] smoothing filter algorithm was used to smooth and denoise the original spectra. Based on comparative evaluations in preliminary pre-experiments, this study set the optimal parameters for SG smoothing to a 15-point window and a 2nd-order polynomial. This method can not only reduce interferences such as detail noise and baseline drift, but also better preserve the inherent characteristics of the spectral curves. To further enhance the absorption and reflection peaks of the soil spectral curves to facilitate the extraction of characteristic bands for soil organic matter, this study applied combinations of three single preprocessing methods—First derivative (FD) [40], Standard normal variate (SNV) [41], and Multiplicative scatter correction (MSC) [42]—to the smoothed spectra. The combined preprocessing methods were denoted as SG+FD, SG+SNV, SG+MSC, SG+FD+SNV, and SG+FD+MSC. All the aforementioned preprocessing algorithms were implemented in Python software (version 3.9).

#### 2.4.2. Construction of Physical Spectral Indices

Spectral indices are a commonly used method for SOM estimation using hyperspectral data. Compared to single wavebands, spectral indices calculated by combining the reflectance of different wavebands not only account for spectral correlations and contain rich soil characteristic information but also improve information utilization to a certain extent [43]. Based on the five preprocessing methods applied to the soil spectra, three physical indices were constructed. The first is the brightness index (BI) [18,44], which reflects the influence of SOM on spectral reflectance. Its calculation formula is as follows:

(1) $$BI=\frac{1}{n}\sum_{i=1}^{n}R_{\lambda i}$$

Secondly, the colour index (CI) [45,46] was employed. It refers to the classical humus ratio and characterizes the color features of organic matter:

(2) $$CI=\frac{R_{465}}{R_{665}}$$

Finally, the normalised difference index (NDI) [47,48] was constructed using the red and near-infrared wavebands, which can enhance the spectral contrast between organic matter and iron oxides:

(3) $$NDI=\frac{R_{850}-R_{670}}{R_{850}+R_{670}}$$

#### 2.4.3. Spectral Feature Band Selection Algorithms

**Figure 3:**
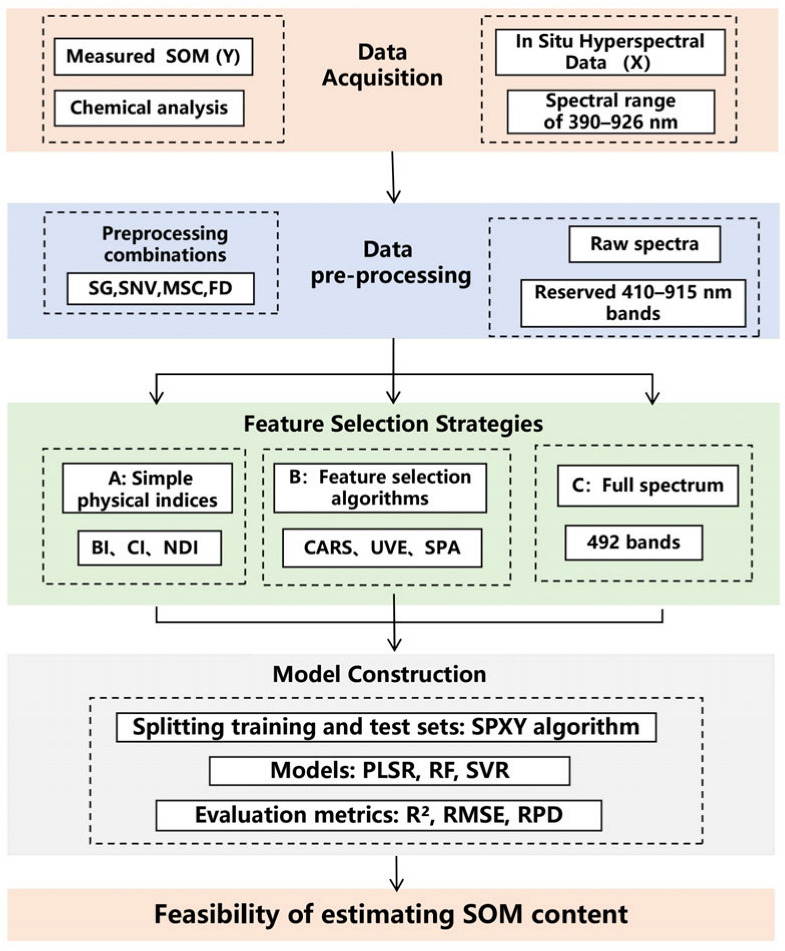
Flowchart of the methodology.

To effectively eliminate the collinearity among waveband variables in the spectral data and reduce data redundancy, this study extracted the characteristic wavebands related to soil organic matter (SOM) using three feature selection methods to improve the computational speed and estimation performance of the models [28]. Concurrently, the workflow of data preprocessing, feature selection, and subsequent model construction is illustrated in Figure 3.

1. Competitive Adaptive Reweighted Sampling (CARS)

The competitive adaptive reweighted sampling (CARS) algorithm [49,50] primarily utilizes an exponentially decreasing function and adaptive reweighted sampling to gradually eliminate variables with minor contributions to the model, retaining only highly adaptable wavebands. Subsequently, the algorithm evaluates the performance of different variable subsets through cross-validation and calculates their root mean square error of cross-validation (RMSECV). Finally, the subset with the minimum RMSECV is selected as the optimal variable combination, ensuring the modeling efficiency and estimation performance.

2. Uninformative Variable Elimination (UVE)

The uninformative variable elimination (UVE) algorithm and [51,52] is a feature selection algorithm based on partial least squares (PLS). Its core idea is to evaluate the regression coefficients and stability of each variable by adding random noise to the data. The algorithm sets a threshold based on the stability of the variables to eliminate those unstable “uninformative variables,” thereby retaining the effective variables that contribute the most to the model. This algorithm can effectively reduce data dimensionality while significantly improving the estimation performance and stability of the model.

3. Successive Projections Algorithm (SPA)

The Successive Projections Algorithm (SPA) [53] is a feature selection method based on forward variable selection, aiming to find a variable subset with minimum collinearity from high-dimensional data. Its core principle involves projecting wavebands onto other wavebands and comparing the magnitudes of the projection vectors, thereby selecting the waveband with the maximum projection vector and subsequently determining the final characteristic wavebands based on the calibration model. This algorithm significantly reduces model complexity and data overlap, effectively mitigating data redundancy and collinearity issues [54].

### 2.5. Model Construction and Accuracy Evaluation

#### 2.5.1. Partial Least Squares Regression (PLSR)

Partial least squares regression (PLSR) is an advanced multivariate statistical analysis method proposed by Wold et al. [55]. This algorithm can effectively extract key spectral information and reduce the collinearity among wavebands, thereby improving the estimation performance and stability of the models; it is particularly suitable for modeling with small sample sizes [56].

#### 2.5.2. Support Vector Regression (SVR)

The Support Vector Regression (SVR) is a data mining method proposed by Cortes and Vapnik [57] that can avoid the problems of local optima and overfitting. By selecting the nonlinear radial basis function (RBF) kernel, the SVR can map low-dimensional nonlinear data into a high-dimensional space for linear analysis. Compared to traditional linear methods, this algorithm reduces model estimation errors [58] and exhibits superior performance in handling complex nonlinear problems such as soil organic matter (SOM) estimation.

#### 2.5.3. Random Forest (RF)

The random forest (RF) algorithm [59] is an ensemble machine learning method based on multiple independent decision trees. Its core advantage lies in its ability to effectively handle the collinearity of spectral data and avoid overfitting, exhibiting stable performance in SOM estimation. In this study, the value range of the mtry parameter in the RF model was set from 1 to the total number of independent variables, and the number of decision trees was set from 10 to 200. These parameters were optimised using a cross-validation strategy, and the RF model yielding the minimum root mean square error RMSE was selected as the final model.

#### 2.5.4. Model Evaluation

During the model construction phase, this study utilized 10-fold cross-validation to avoid random bias caused by sample partitioning. Meanwhile, the core parameters of each feature selection algorithm and estimation model are shown in Table 2. Meanwhile, the model evaluation metrics used included the coefficient of determination (R^2^), root mean square error (RMSE), and residual prediction deviation (RPD). R^2^ reflects the fitting degree of the model, which is the model’s ability to explain the variation in the dependent variable [60]. An R^2^ value closer to 1 indicates a better fitting effect and higher stability of the model. RMSE represents the average error between the predicted values and the actual values of the model [61]. A smaller RMSE value indicates a higher prediction accuracy and a stronger estimation capability of the model. RPD is the ratio of the sample standard deviation to the RMSE, used to evaluate the predictive ability of the model. When RPD < 1.4, the model lacks predictive ability; when 1.4 ≤ RPD < 2.0, the model has a rough estimation ability; when RPD ≥ 2.0, the model has good predictive ability [62]. The relevant formulas are as follows:

(4) $$R^{2}=\frac{\sum_{i=1}^{n}{\left({\hat{y}}_{i}-{\bar{y}}_{i}\right)}^{2}}{\sum_{i=1}^{n}{\left(y_{i}-{\bar{y}}_{i}\right)}^{2}}$$

(5) $$RMSE=\sqrt{\frac{1}{n}\sum_{i=1}^{n}{\left({\hat{y}}_{i}-y_{i}\right)}^{2}}$$

(6) $$RPD=\frac{SD}{\mathrm{RMSE}}$$

where *n* is the total number of soil samples, *y_i_* is the measured value of the soil fertility attribute content, *ŷ_i_* is the predicted value, and ${\bar{y}}_{i}$ is the mean measured value of the soil fertility attribute content.

## 3. Results

### 3.1. Descriptive Statistics of Soil Organic Matter Content

The descriptive statistics of the SOM content in the study area are presented in Table 3. After the removal of outliers, the SOM content ranged from 1.02 to 42.49 g·kg^−1^. The mean values of the total samples, calibration set, and validation set were 15.75 g·kg^−1^, 15.66 g·kg^−1^, and 16.00 g·kg^−1^, respectively, with corresponding coefficients of variation (CV) of 68.89%, 69.15%, and 69.08%. According to the statistical results and relevant studies, the differences between the calibration set and validation set can enhance model prediction accuracy, with the average values and coefficient of variation in total samples calculated between the calibration and validation sets meeting modelling standards.

### 3.2. Characteristics of Soil Spectral Curves Under Different Pre-Processing Methods

**Figure 4:**
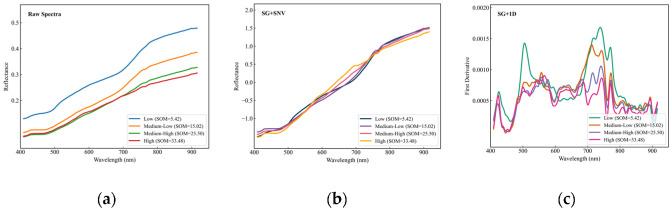
Spectral reflectance curves of soil samples with different soil organic matter (SOM) contents: (**a**) Raw spectra; (**b**) SG+SNV spectra; (**c**) SG+1D spectra.

For the 139 soil samples, their organic matter contents were sorted from lowest to highest and divided into four groups: 1–10 g/kg, 10–20 g/kg, 20–30 g/kg, and 30–40 g/kg. The average value for each group was calculated, and the spectral curve patterns of the organic matter samples in different groups were analyzed, as shown in Figure 4. The results indicated that there were certain differences among the soil spectral curves with varying organic matter contents, but their variation trends with wavelength were similar. An analysis of the raw spectral curves for different organic matter content levels (Figure 4a) revealed that within the 409–915 nm range, all four average spectral curves showed a slowly rising trend, and the spectral reflectance increased as the wavelength increased. Meanwhile, the spectral curves for organic matter contents of 33.48 g/kg and 25.50 g/kg intersected and overlapped within the 610–710 nm range. In the 682–915 nm range, a negative correlation was observed between the soil organic matter content and the spectral curves, indicating that as the soil organic matter content increased, the spectral reflectance tended to decrease. This phenomenon objectively reflects the strong light absorption by dark humic substances in SOM.

Within the limited response range of the visible-near-infrared (Vis-NIR) spectra, compared to the raw spectra, the preprocessing methods altered the original morphological characteristics of the spectra. After the SG+SNV processing (Figure 4b), the variation patterns in the raw spectra caused by different organic matter contents were completely eliminated, causing the spectral curves of the different groups to intertwine and overlap. Due to the low signal-to-noise ratio of the field in situ spectral data, although the spectral curves after SG+1D processing (Figure 4c) enhanced local absorption peaks, they also amplified high-frequency noise to a certain extent. This indicates that, limited by factors such as the restricted band range and the quality of the raw spectral data, conventional preprocessing methods may have a negative impact on the models.

### 3.3. Selection of Sensitive Characteristic Bands for Soil Organic Matter

**Figure 5:**
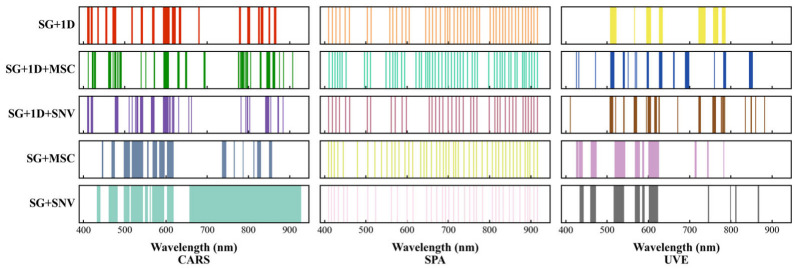
Distribution of characteristic bands under different feature variable selection algorithms.

To further determine the sensitive spectral bands of soil organic matter (SOM), this study applied the CARS, UVE, and SPAs to extract features from the raw and preprocessed spectra, and the extraction results are illustrated in Figure 5. After extraction by the three algorithms, over 70% of the bands were eliminated. The CARS algorithm retained the highest number of characteristic bands, accounting for 17.1–31.16% of the total number of bands, followed by the UVE and SPAs, which accounted for 11–20.37% and 1.22–11.20%, respectively. Most of the sensitive bands extracted by the CARS and UVE algorithms were distributed within the 500–650 nm range. Among them, the bands extracted by the CARS algorithm exhibited obvious clustering within the 500–650 nm range, and the sensitive bands extracted by the UVE algorithm also showed a similar pattern. This indicates that the algorithm can effectively identify the characteristic bands related to organic matter; in particular, the features affected by the electron transition responses of iron oxide and SOM complexes in the soil are mainly distributed within the 500–650 nm range. Its combination with the SG+SNV algorithm resulted in the highest proportion of extracted bands (67.03%); the SPAs extracted the fewest characteristic bands (15.38%), exhibiting a sparse and uniformly distributed state, which might even lead to the loss of relevant signal features.

### 3.4. Comparison of SOM Estimation Results Under Different Feature Strategies and Models

Table 4 systematically presents the estimation performance of the soil organic matter prediction models on the calibration and prediction sets under different strategies (see Appendix A for complete results). Overall, the models built based on the raw spectra exhibited better estimation performance than those subjected to SG+SNV preprocessing. After SG+SNV preprocessing, the estimation performance of most models exhibited a substantial decrease. Taking the RF model as an example, after the Group A strategy was processed with SG+SNV, its prediction set RPD decreased from 2.18 to 1.01. The estimation performance of the models built with the Group B and Group C strategies also decreased by 0.25 and 0.75, respectively, and some models even lost the capability to effectively predict SOM. These results further indicate that the preprocessing algorithms have a negative impact on the estimation performance of organic matter.

Among the effective models built based on the raw spectra, different combinations of feature extraction strategies and algorithms exhibited distinct performance differences. In particular, the CARS-SVR model in Group B exhibited the best overall prediction performance, with the R^2^, RMSE, and RPD of the prediction set reaching 0.83, 4.29 g/kg, and 2.45, respectively. This indicates that this feature extraction strategy can effectively eliminate redundant bands and capture weak effective spectral information. The estimation performance (RPD = 2.30) of Group C, which directly utilized the full-spectrum data combined with the SVR model, was slightly lower than that of Group B, indicating that the redundant information present in the full bands caused certain interference with the upper limit of the estimation. Notably, Group A also achieved stable estimation performance (RPD = 2.18) relying solely on the combination of three simple physical indices (BI, CI, NDI) and the RF model, demonstrating that the low-cost modeling strategy using physical indices holds favorable application potential in this study area.

Furthermore, comparing the estimation performance between the calibration and prediction sets of each model reveals that the models based on raw spectra exhibited reliable estimation performance. In particular, the Group B CARS-SVR model, which had the best overall performance, maintained robust estimation performance from its calibration set (R^2^ = 0.69) to its prediction set (R^2^ = 0.83). This indicates that the CARS-SVR algorithm can extract effective characteristic band information and achieve dimensionality reduction without exhibiting overfitting, demonstrating favorable robustness and stability.

### 3.5. Error Analysis of Different Models Across Various SOM Content Ranges

To evaluate the prediction stability and error variability of the models across different SOM content ranges, and to reveal the compatibility between feature extraction strategies and modeling algorithms, this study selected three representative models (Group A Raw-RF, Group B Raw-CARS-SVR, and Group C Raw-SVR). Scatter plots of the measured versus predicted values for the test set samples were generated to conduct a comparative analysis from three dimensions: fitting performance, residual distribution, and segmented estimation performance.

**Figure 6:**
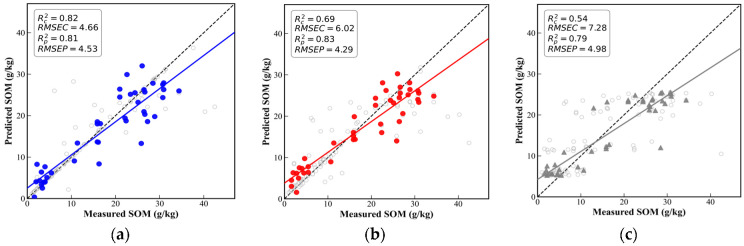
Scatter fitting characteristics of global SOM estimation under different feature-model combinations: (**a**) SVR; (**b**) CARS-SVR; (**c**) RF.

As shown in Figure 6, the predicted SOM content values of the three models were closely distributed around the 1:1 line within the 0–5 g/kg range. In particular, the predicted values of the Group B Raw-CARS-SVR model were the most convergent, exhibiting no severe deviation points (R^2^ = 0.83, RMSE = 4.29). The Group C Raw-SVR model, without feature extraction, exhibited acceptable overall estimation performance, but clear divergence occurred in the high-content range where SOM > 25 g/kg. The slope of the Group A Raw-RF model was noticeably lower than the 1:1 line, and the predicted SOM values in the high-content region were generally located below the diagonal, exhibiting poor overall prediction stability.

**Figure 7:**
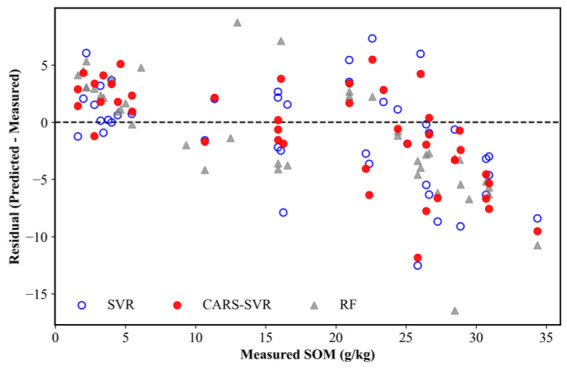
Distribution of estimation residuals versus SOM content under different matching strategies.

To intuitively reveal the prediction uncertainty, this study further analyzed the variations in residuals with SOM content for the three models (Figure 7), revealing that the residual distribution exhibited an underestimation phenomenon at high values. When SOM < 10 g/kg, all models exhibited slight positive residuals. When SOM > 25 g/kg, the residuals of all models began to decline markedly; in particular, the residual of the Group A Raw-RF model fell below −10 g/kg.

**Figure 8:**
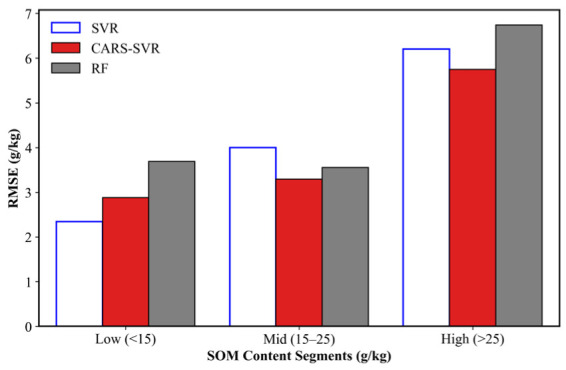
Segmented RMSE of different models for SOM content estimation.

To further quantify the estimation stability of the aforementioned models across different SOM content ranges, Figure 8 and Table 5 detail and compare the segmented RMSE performance of the models in three SOM intervals: low (<15 g/kg), medium (15–25 g/kg), and high (>25 g/kg). Overall, the errors of each model exhibited distinct phased differences as SOM content increased.

In the low-content interval (SOM < 15 g/kg), all three groups of strategies maintained low prediction errors. Among them, the full-spectrum SVR model in Group C exhibited the lowest error, with an RMSE of 2.34 g/kg; however, Group A, relying solely on the combination of three simple physical indices and the RF model, also achieved a favorable estimation performance (RMSE = 3.69 g/kg). In the medium-content interval (15–25 g/kg), the error performance of each model remained relatively stable, with the Raw-CARS-SVR model in Group B achieving the optimal local estimation performance within this interval (RMSE = 3.29 g/kg).

However, when the SOM content exceeded 25 g/kg, the differences in estimation performance among the models gradually increased. The errors of the Group A Raw-RF and Group C Raw-SVR models rose progressively, with their RMSE values reaching 6.74 g/kg and 6.20 g/kg, respectively. In contrast, the error level of the Group B Raw-CARS-SVR model remained relatively low at 5.75 g/kg. When the models built based on the feature sets of Group A and Group C exhibited larger errors in the high SOM content interval, the Group B strategy still maintained stable estimation performance, demonstrating favorable anti-interference capability and robustness.

## 4. Discussion

### 4.1. Spectral Response Mechanisms and the Impacts of Pre-Processing

Existing studies have shown that preprocessing methods can, to some extent, mitigate background noise introduced by measuring instruments, analytical methods, and environmental factors [21]. However, this study found that after applying preprocessing techniques, including scatter correction (SG+MSC, SG+SNV) and combined derivative transformations (e.g., SG+1D, SG+1D+SNV, SG+1D+MSC), the estimation performance of the SOM models was inferior to those based on the raw soil spectra (Table 4). This indicates that under the specific constraints of the limited visible and shortwave near-infrared (390–926 nm) spectral range and the inherent low signal-to-noise ratio of the field in situ spectral data, conventional preprocessing methods exerted a negative impact on the SOM estimation in Moso bamboo forests. In the 450–800 nm range, the spectral response of soil organic matter (SOM) is primarily driven by electron transitions [16,63], which macroscopically manifests as variations in overall soil color. Particularly in the Moso bamboo forest soils of southern regions, the spectral features within this wavelength range reflect not only the darkening effect of SOM, but are also jointly influenced by the strong electron transition absorption of iron oxides and residual soil moisture. A higher SOM content (Figure 4a) exhibits a stronger light absorption capacity, leading to an overall decrease in spectral reflectance and a gradual darkening of the soil color [16]. On the spectral curves, this effect is characterized by hierarchical differences in overall reflectance. However, the principles of the MSC and SNV algorithms primarily rely on utilizing normalized spectral intensity to correct for multiplicative scattering and baseline drift [42,64]. Although the first derivative and its combined preprocessing methods [40] can theoretically separate overlapping absorption peaks effectively, they are highly prone to amplifying the inherent high-frequency background noise of the instrument within a limited spectral range (Figure 4c). This leads to a decreased signal-to-noise ratio, thereby masking the weak signal features associated with SOM [65]. These results demonstrate that while these preprocessing methods eliminate physical noise, they may concurrently attenuate the feature signals associated with SOM, such as color and brightness [17,18]. Consequently, the original hierarchical differences in the reflectance spectral curves among soil samples with varying SOM contents are eliminated, resulting in intertwined and overlapping spectral profiles (Figure 4b). Therefore, for the SOM estimation in Moso bamboo forests, preserving the integrity of the weak spectral signal features proves to be more critical than eliminating the multiplicative scattering effects of the soil spectra.

### 4.2. Matching Between Different Feature Selection Algorithms and Models

Numerous studies have demonstrated that feature extraction algorithms can effectively overcome collinearity and noise interference in soil spectra [54], retaining core features while eliminating redundant information. When extracting effective features, the CARS algorithm outperformed UVE and SPA (Figure 5), which is primarily attributed to their distinct selection mechanisms [52,53]. The spectral features within the 400–915 nm range are predominantly broad and continuous curves (Figure 4) rather than sharp absorption peaks. However, because the SPAs focuses on extracting the characteristic peaks or troughs with the greatest variations [66], it leads to the loss of most spectral background information related to SOM, subsequently causing model underfitting. This aligns with the findings of Vohland et al. [45]. In contrast, the CARS algorithm emphasizes the interactions among variables; while filtering out redundant information, it effectively retains the continuous feature subsets associated with SOM, thereby enhancing the overall estimation performance [49].

This study found that, building upon the extraction of effective spectral features and considering the strong spatial heterogeneity of soil organic matter (SOM) in Moso bamboo forests, coupling different feature extraction strategies with compatible modeling algorithms can effectively resolve the estimation difficulties across different SOM content intervals. When the SOM content falls within the low-content interval (SOM < 15 g/kg), the suboptimal estimation performance is primarily attributed to weak effective spectral signals and their susceptibility to high-frequency background noise. As indicated in Table 4, the RPD of the RF model based on the three features from Group A reached 2.18; however, when inputting the 128 features extracted by CARS in Group B, the RPD paradoxically dropped by 0.55. Fundamentally, the three simple physical indices (BI, CI, and NDI) in Group A enable the RF model to directly focus on signal features such as soil color and brightness [67]. Given the limited sample size, this effectively prevents the RF model from suffering from noise interference or falling into overfitting when processing high-dimensional data [8]. Conversely, when estimating SOM content using the feature sets from Group B and Group C combined with the RF algorithm, the model is prone to selecting suboptimal or noisy bands for prediction, thereby triggering overfitting. This demonstrates that the Group A strategy (simple physical indices) coupled with the RF model exhibits favorable adaptability within this specific low-content interval.

When the SOM content falls within the high-content interval (SOM > 25 g/kg), single-model algorithms exhibited a pronounced underestimation of high values (Figure 7). This is likely because the SOM content in the study area is predominantly distributed within the medium-content interval, causing the models to lean towards fitting the characteristic patterns of the majority of samples. Consequently, as the soil SOM content increases, the model’s sensitivity to SOM features diminishes. Nevertheless, the synergistic estimation using the CARS algorithm from the Group B strategy combined with the SVR model effectively reduced the estimation error within this specific interval (Figure 8) and maintained a relatively reliable estimation level. By extracting 128 features, the CARS algorithm successfully retains weak spectral details [49]. Leveraging its inherent advantage in handling nonlinear relationships within medium- to high-dimensional feature spaces, the SVR algorithm utilizes the radial basis function (RBF) kernel to map this weak information into a high-dimensional space, thereby constructing an optimal separating hyperplane [58]. The synergistic combination of these two algorithms fully capitalizes on the weak soil spectral features, achieving the optimal estimation performance for SOM content in Moso bamboo forests (R^2^ = 0.83, RMSE = 4.29, RPD = 2.45). These results demonstrate that the proposed strategies can serve as a valuable reference for the rapid estimation of soil organic matter in Moso bamboo forests.

Although the proposed estimation strategies have demonstrated effectiveness in SOM estimation within Moso bamboo forests, the current sample size remains relatively limited and is confined to a single geographic region due to the inherent challenges of field in situ sampling. Given these experimental constraints, this study prioritized conventional machine learning algorithms for constructing the estimation models, and external validation using an independent dataset has not yet been conducted. Future research will consider incorporating deep learning algorithms to construct estimation models, alongside verifying their stability and transferability across diverse soil types and full-range spectral conditions.

## 5. Conclusions

This study investigated Moso bamboo forest soils based on field in situ spectral data and measured soil organic matter (SOM) data, exploring the synergistic adaptation strategies coupling different preprocessing methods, feature extraction strategies, and machine learning algorithms for estimating SOM in Moso bamboo forests. The main conclusions are as follows: (1) Within the limited visible and shortwave near-infrared (Vis-NIR) wavelength range, the majority of preprocessing algorithms applied to raw field spectra exerted a negative impact on SOM estimation. While mitigating the multiplicative scattering effects of soil spectra, these algorithms concurrently disrupted the feature signals associated with SOM, leading to a severe decline in model performance. Therefore, preserving the integrity of the spectral signal and utilizing raw spectra to estimate SOM proved to be a more reliable estimation method in this study. (2) For Moso bamboo forest soils with extreme spatial heterogeneity, estimating different SOM contents requires matching appropriate models; that is, there is distinct adaptability when combining different feature extraction strategies with modeling algorithms. Within the low-content interval (<15 g/kg), the Group A approach—utilizing simple physical indices based on raw wavebands combined with the RF model—demonstrated good estimation potential and low computational cost (R^2^ = 0.79, RMSE = 4.99, RPD = 2.18), achieving effective SOM estimation at a minimal computational expense. When estimating SOM in the high-content interval (SOM > 25 g/kg), the Group B approach, coupling the SVR model with the CARS algorithm, yielded the optimal estimation performance (R^2^ = 0.83, RMSE = 4.29, RPD = 2.45). This approach effectively reduced the underestimation of high values caused by sample distribution imbalance. This study not only clarified the spectral response mechanisms of SOM within a limited wavelength range but also validated the effectiveness and feasibility of the feature extraction algorithms and model synergistic adaptation strategy for estimating SOM in Moso bamboo forests under current experimental conditions. The proposed approach can serve as a certain reference for SOM monitoring in this specific region.

## Figures and Tables

**Table 1:** Summary of basic site characteristics and management practices in the sampling plots.

Variables	Sampling Extent per Plot	Intra-Plot Distance	Elevation (m)	Slope	Aspect	Microrelief	Stand Age Structure	Soil Types	Management
Characteristics/Description	30 m × 30 m	10–15 m	664.0–892.0 m	10.7–39.0°	South, Southeast, and Northwest	upper, middle, lower slope	uneven-aged mixed structure: 1-du, 2-du, 3-du, and 4-du bamboo	yellow soil and red soil	Biennial fertilization and weeding

**Table 2:** Key parameters and hyperparameter search spaces for feature selection algorithms and estimation models.

Stage	Algorithm/Model	Key Parameters/Search Space
Feature Selection	CARS	Monte Carlo runs = 100
	UVE	Stability threshold= 0.8; Adaptive noise = 1% of global STD
	SPA	Max selected variables = 60;
Estimation Models	SVR	C ∈ [0.1, 1000]; γ ∈ [10^−4^, 0.1]
	RF	n_estimators ∈ [50, 300]; max_depth ∈ [3, 10]
	PLSR	n_components ∈ [1, 15]

**Table 3:** Descriptive Statistics of Soil Organic Matter Content.

Total	Samples	Max (g/kg)	Min (g/kg)	Mean (g/kg)	SD (g/kg)	CV (%)
total samples	139	42.49	1.02	15.75	10.85	68.89
calibration set	97	42.49	1.40	15.66	10.83	69.15
validation set	42	34.36	1.02	16.00	11.02	69.08

**Table 4:** Estimation performance of soil organic matter (SOM) using different machine learning models under various feature-model strategies.

Model	Strategy	Data Type	Calibration Set	Prediction Set
			R^2^/RMSE	R^2^/RMSE/RPD
PLSR	A (Simple Physical Indices)	Indices (Raw)	0.41/8.28	0.71/5.86/1.86
		Indices (SG+SNV)	0.09/10.58	0.04/9.78/1.02
	B (Feature Selection)	CARS (Raw)	0.57/7.05	0.73/5.43/1.94
		CARS (SG+SNV)	0.69/6.07	0.70/5.59/1.82
	C (Full Spectrum)	Raw	0.72/5.76	0.46/7.62/1.37
		SG+SNV	0.71/5.89	0.61/6.18/1.60
SVR	A (Simple Physical Indices)	Indices (Raw)	0.47/7.81	0.77/5.20/2.09
		Indices (SG+SNV)	0.14/10.30	0.05/9.73/1.03
	B (Feature Selection)	CARS (Raw)	**0.69/6.02**	**0.83/4.29/2.45**
		CARS (SG+SNV)	0.61/6.84	0.66/5.92/1.72
	C (Full Spectrum)	Raw	**0.82/4.66**	**0.81/4.53/2.30**
		SG+SNV	0.51/7.67	0.51/7.77/1.27
RF	A (Simple Physical Indices)	Indices (Raw)	**0.54/7.27**	**0.79/4.99/2.18**
		Indices (SG+SNV)	0.46/8.20	0.02/9.84/1.01
	B (Feature Selection)	CARS (Raw)	0.69/5.98	0.62/6.44/1.63
		CARS (SG+SNV)	0.54/7.41	0.48/7.38/1.38
	C (Full Spectrum)	Raw	0.63/6.65	0.76/5.05/2.06
		SG+SNV	0.69/6.13	0.42/7.55/1.31

**Table 5:** Quantitative statistics of segmented RMSE for different matching strategies.

SOM Content Interval (g/kg)	Strategy A: Raw-RF RMSE (g/kg)	Strategy B: Raw-CARS-SVR RMSE (g/kg)	Strategy C: Raw-SVR RMSE (g/kg)
Low-content (<15)	3.69	2.88	2.34
Medium-content (15–25)	3.56	3.29	4.00
High-content (>25)	6.74	5.75	6.20

## Data Availability

The data presented in this study are available on request from the corresponding author. The data are not publicly available due to privacy and ongoing research restrictions.

## Abbreviations

The following abbreviations are used in this manuscript:

SOM	Soil Organic Matter
Vis-NIR	Visible and Near-Infrared
PLSR	Partial Least Squares Regression
RF	Random Forest
SVR	Support Vector Regression
CARS	Competitive Adaptive Reweighted Sampling
UVE	Uninformative Variable Elimination
SPA	Successive Projections Algorithm
SPXY	Sample Set Partitioning Based on Joint X-Y Distances
BI	Brightness Index
CI	Color Index
NDI	Normalized Difference Index
SG	Savitzky–Golay
SNV	Standard Normal Variate
MSC	Multiplicative Scatter Correction
FD	First Derivative
R^2^	Coefficient of Determination

## Appendix A

Table A1, Table A2 and Table A3 present the comprehensive SOM content estimation results across all combinations of the five spectral pre-processing methods, three feature selection algorithms, and three machine learning models (PLSR, SVR, and RF). These exhaustive results supplement the focused comparative analysis presented in the main text.

**Table A1:** Estimation results of SOM content based on the PLSR model.

Model	Feature	Pre- Processing	No. of Bands	Training Set		Test Set		
				R^2^	RMSE	R^2^	RMSE	RPD
PLSR	Indices (BI, CI, NDI)	R	3	0.41	8.28	0.71	5.86	1.86
		SG+1D	3	0.13	10.43	0.29	8.28	1.18
		SG+MSC	3	0.06	10.69	0.05	9.99	1.03
		SG+SNV	3	0.09	10.58	0.04	9.78	1.02
		SG+1D+MSC	3	0.02	10.95	0.04	9.95	1.02
		SG+1D+SNV	3	0.01	10.44	−0.26	11.90	0.89
	CARS	R	128	0.57	7.05	0.73	5.43	1.94
		SG+1D	93	0.55	7.24	0.51	6.49	1.44
		SG+MSC	119	0.72	5.87	0.66	5.67	1.72
		SG+SNV	153	0.69	6.07	0.70	5.59	1.82
		SG+1D+MSC	84	0.74	5.68	0.62	6.30	1.62
		SG+1D+SNV	93	0.62	6.77	0.59	6.65	1.56
	UVE	R	85	0.52	7.43	0.72	5.67	1.88
		SG+1D	69	0.57	7.11	0.55	6.20	1.49
		SG+MSC	100	0.59	7.13	0.65	5.73	1.68
		SG+SNV	91	0.68	6.28	0.62	6.06	1.62
		SG+1D+MSC	54	0.61	6.90	0.59	6.52	1.56
		SG+1D+SNV	56	0.67	6.24	0.65	6.04	1.70
	SPA	R	6	0.42	8.27	0.65	6.01	1.70
		SG+1D	47	0.55	7.22	0.53	6.70	1.46
		SG+MSC	48	0.71	5.93	0.65	6.06	1.68
		SG+SNV	38	0.47	7.96	0.63	6.20	1.65
		SG+1D+MSC	55	0.28	9.35	0.205	9.25	1.12
		SG+1D+SNV	39	0.56	7.18	0.70	5.90	1.84
	None	R	492	0.72	5.76	0.46	7.62	1.37
		SG+1D	492	0.56	7.12	0.36	7.20	1.25
		SG+MSC	492	0.74	5.58	0.66	5.89	1.71
		SG+SNV	492	0.71	5.89	0.61	6.18	1.60
		SG+1D+MSC	492	0.30	9.30	0.29	8.50	1.18
		SG+1D+SNV	492	0.56	7.18	0.71	5.83	1.87

**Table A2:** Estimation results of SOM content based on the SVR model.

Model	Feature	Pre- Processing	No. of Bands	Training Set		Test Set		
				R^2^	RMSE	R^2^	RMSE	RPD
SVR	Indices (BI, CI, NDI)	R	3	0.47	7.81	0.77	5.20	2.09
		SG+1D	3	0.31	9.24	0.33	8.01	1.22
		SG+MSC	3	0.11	10.38	0.06	9.89	1.03
		SG+SNV	3	0.14	10.30	0.05	9.73	1.03
		SG+1D+MSC	3	−0.04	11.29	−0.02	10.24	0.98
		SG+1D+SNV	3	0.13	9.80	−0.67	13.73	0.77
	CARS	R	128	0.69	6.01	0.83	4.29	2.45
		SG+1D	93	0.79	4.91	0.43	7.04	1.32
		SG+MSC	119	0.52	7.64	0.50	6.89	1.41
		SG+SNV	153	0.61	6.84	0.66	5.92	1.72
		SG+1D+MSC	84	0.74	5.61	0.65	5.96	1.70
		SG+1D+SNV	93	0.60	6.87	0.71	5.51	1.87
	UVE	R	85	0.53	7.39	0.78	4.91	2.18
		SG+1D	69	0.71	5.89	0.64	5.56	1.66
		SG+MSC	100	0.56	7.43	0.60	6.13	1.57
		SG+SNV	91	0.56	7.36	0.67	5.62	1.74
		SG+1D+MSC	54	0.82	4.74	0.58	6.60	1.55
		SG+1D+SNV	56	0.73	5.66	0.78	4.98	2.13
	SPA	R	6	0.46	8.01	0.68	5.80	1.75
		SG+1D	47	0.71	5.81	0.59	6.27	1.55
		SG+MSC	48	0.54	7.45	0.50	7.22	1.41
		SG+SNV	38	0.48	7.89	0.58	6.61	1.55
		SG+1D+MSC	55	0.66	6.38	0.53	7.07	1.46
		SG+1D+SNV	39	0.50	7.61	0.59	6.95	1.56
	None	R	492	0.82	4.66	0.81	4.53	2.30
		SG+1D	492	0.74	5.54	0.48	6.51	1.38
		SG+MSC	492	0.55	7.38	0.43	7.63	1.32
		SG+SNV	492	0.51	7.67	0.38	7.77	1.27
		SG+1D+MSC	492	0.78	5.20	0.68	5.66	1.78
		SG+1D+SNV	492	0.80	4.82	0.78	5.14	2.12

**Table A3:** Estimation results of SOM content based on the RF model.

Model	Feature	Pre- Processing	No. of Bands	Training Set		Test Set		
				R^2^	RMSE	R^2^	RMSE	RPD
RF	Indices (BI, CI, NDI)	R	3	0.54	7.27	0.79	4.99	2.18
		SG+1D	3	0.31	9.25	0.40	7.60	1.30
		SG+MSC	3	0.26	9.46	0.14	9.53	1.07
		SG+SNV	3	0.46	8.20	0.02	9.84	1.01
		SG+1D+MSC	3	0.08	10.60	0.15	9.32	1.08
		SG+1D+SNV	3	0.16	9.59	0.14	12.58	0.84
	CARS	R	128	0.69	5.98	0.62	6.44	1.63
		SG+1D	93	0.84	4.22	0.41	7.13	1.30
		SG+MSC	119	0.72	5.88	0.55	6.56	1.49
		SG+SNV	153	0.54	7.41	0.48	7.38	1.38
		SG+1D+MSC	84	0.84	4.32	0.51	7.10	1.43
		SG+1D+SNV	93	0.89	3.61	0.73	5.33	1.94
	UVE	R	85	0.84	4.24	0.70	5.83	1.83
		SG+1D	69	0.88	3.72	0.48	6.66	1.39
		SG+MSC	100	0.76	5.48	0.59	6.16	1.56
		SG+SNV	91	0.82	4.73	0.62	6.09	1.61
		SG+1D+MSC	54	0.69	6.11	0.51	7.13	1.43
		SG+1D+SNV	56	0.85	4.25	0.53	7.05	1.46
	SPA	R	6	0.85	4.19	0.67	5.89	1.73
		SG+1D	47	0.89	3.61	0.41	7.46	1.30
		SG+MSC	48	0.58	7.11	0.57	6.73	1.52
		SG+SNV	38	0.59	6.98	0.55	6.84	1.50
		SG+1D+MSC	55	0.75	5.45	0.56	6.82	1.52
		SG+1D+SNV	39	0.87	3.82	0.76	5.32	2.04
	None	R	492	0.63	6.65	0.76	5.05	2.06
		SG+1D	492	0.83	4.43	0.34	7.33	1.29
		SG+MSC	492	0.63	6.76	0.48	7.20	1.38
		SG+SNV	492	0.69	6.13	0.42	7.55	1.31
		SG+1D+MSC	492	0.86	4.14	0.69	5.60	1.79
		SG+1D+SNV	492	0.72	5.72	0.70	6.00	1.81
